# Case report of an internal granuloma investigated by light and scanning electron microscopy

**DOI:** 10.1186/s13005-015-0077-6

**Published:** 2015-06-12

**Authors:** Georg Gassmann, Wolfgang H. Arnold

**Affiliations:** PraxisHochschule Cologne, Dental Hygiene and Preventive Management, Köln, Germany; Department of Biological and Material Sciences in Dentistry, Witten/Herdecke University, Faculty of Health, School of Dentistry, Alfred-Herrhausen-Strasse 44, 58455 Witten, Germany

**Keywords:** Internal granuloma, Root resorption, Pulp inflammation

## Abstract

There is no doubt that the main reason for an internal grauloma is a traumatic event. The trauma may be physical or chemical as in the case of caries or coronal pulpectomy. In most of the cases it is diagnosed by hazard or, when in case of fracture or mobility, extraction is the only therapy to be performed. If diagnosed in time root canal treatment may be adequate.

In the presented case no single specific event could be determined being the cause of this large internal granuloma extending from the coronal third of the root canal to the whole crown just leaving an eggshell of enamel that fractured and mimicked mobility of the whole tooth to the patient finally causing him to attend the clinic. As the patient presented severe aggressive periodontitis and mobility of all teeth it first was assumed that periodontitis was the ethiological reason in this case. Due to secondary trauma the front teeth were labially positioned thus probably being exposed to traumatic insults more frequently. Clinically the upper right medial incisor appeared discoloured darkly not showing the typical pink spot. Without any force the coronal part of the right medial incisor could be removed manually and the root was extracted using a periostal extractor. As it was not suitable to leave the patient with a missing tooth in the front the wound was sutured and as a temporary solution the tooth was reconstructed with composite intraorally and fixed to the neighbour teeth adhesively. The histopathology of the internal granuloma and the crown was investigated.

## Background

Internal pulp granuloma is a very rare disease that is usually caused by traumata. According to Schroeder [1], internal granuloma is a very rare phenomenon with a prevalence of 0.1-1.6 %. Reports about internal pulpal granuloma and internal dentin resorption are found scarcely in the literature [2–4]. Additionally, there is no clear knowledge of the etiology of this disease [5]. Trauma, chronic inflammation of the dental pulp, direct pulp capping and orthodontic tooth movements seem to be the most frequent reasons of its occurrence [6]. In most cases, it is diagnosed by hazard or in case of fracture or mobility. Extraction is the only therapy to be performed.

**Fig. 1:**
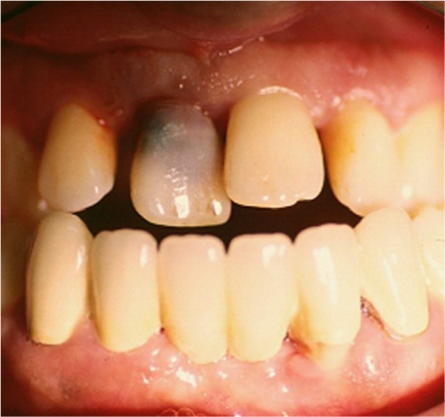
Clinical state of tooth 11 prior to treatment. The tooth shows discoloration

In the case presented here, no single specific event could be identified as being the cause of this large internal granuloma. It extended from the coronal third of the root canal into the whole crown, just leaving an eggshell of enamel that fractured within the gingival attachment border. Thus, mobility of the whole tooth was mimicked to the patient perception, finally causing him to attend the clinic. Because the 23-year-old patient presented with a severe generalized aggressive periodontitis, showing mobility in all of his teeth, this diagnosis was assumed to be the etiological reason. Due to secondary trauma, the front teeth were positioned labially, thus probably being exposed to traumatic insults more frequently. Clinically, the upper right medial incisor appeared discolored darkly, and not showing the typical pink spot (Fig. 1.).

**Fig. 2:**
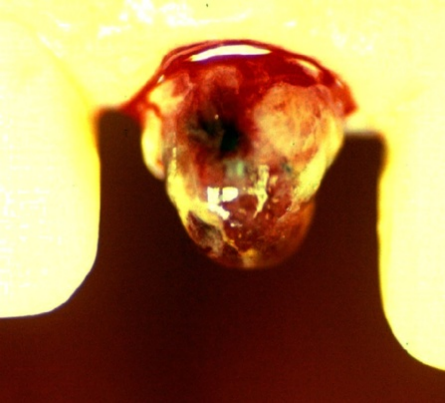
Tooth 11 after manual removal of the crown. A large internal granuloma could be seen

The coronal part of the right medial incisor was very carefully removed, so as not to destroy the remnants of the crown. After removal of the crown, a large internal granuloma was visible (Fig. 2). The root was extracted using a periosteal extractor. As it was not suitable to leave the patient with a missing tooth in the front, the wound was sutured, and as a temporary solution, the tooth was rebuilt with composite by attaching to the neighboring teeth via adhesive. As it was considered to be an extraordinary case in terms of the extension of the internal granuloma, photo documentation was performed. Histological reports together with SEM investigations of internal granulomas are quite rare. Therefore, the root containing pulp and the internal granuloma was prepared to be studied via light microscopy, and the coronal part was prepared for SEM investigation.

## Materials and methods

The coronal part of the tooth was stored in 0.7 % NaCl containing 0.1 % thymol. After dehydration in graded acetone, the crown was critical point dried and sputtered with gold-palladium. Resorption patterns of the inner surface of the crown were studied with a scanning electron microscope (Philips XL 30 FEG) at 20 kV beam current. The surface structure was described in terms of its roughness, regularity/irregularity, structure and texture.

The root with the internal granuloma was fixed in 3.5 % formalin and prepared for light microscopy by decalcification in 5 % HNO_3_, sectioning after embedding in paraffin, and then staining with azan. According to the light microscopic investigation of the internal granuloma cell types, the composition and structure of the resorbing granulation tissue were described.

## Results

**Fig. 3:**
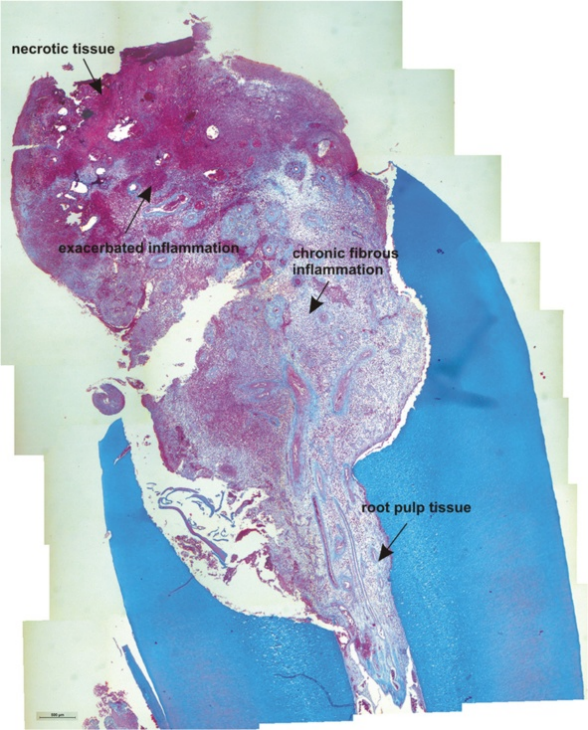
Histological section of the root with internal granuloma. Four different zones could be distinguished: 1. necrosis, 2. exacerbated inflammation, 3. chronic inflammation, 4. approximately normal root pulp tissue

**Fig. 4:**
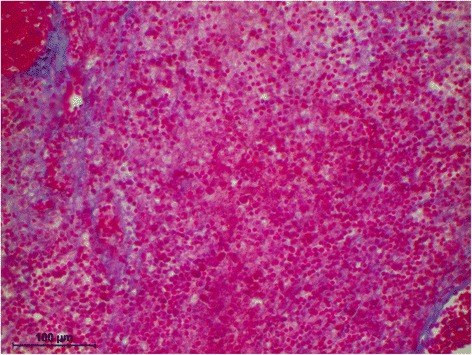
Higher magnification of the area with exacerbated inflammation, exhibiting fibrous sheets around the blood vessels

**Fig. 5:**
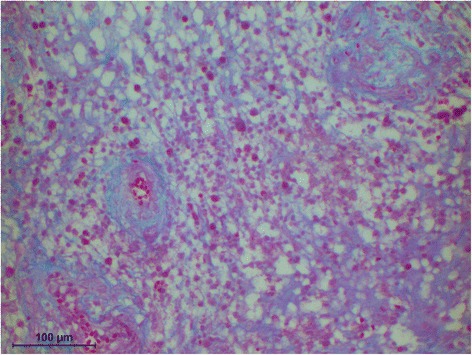
Higher magnification of the area with chronic inflammation, exhibiting fibrous sheets around the blood vessels Higher magnification of the pulp dentin border with odontoclasts in resorption lacunae

**Fig. 6:**
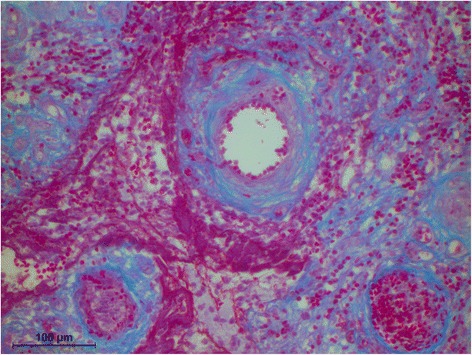
Fibrous sheet around blood vessels

**Fig. 7:**
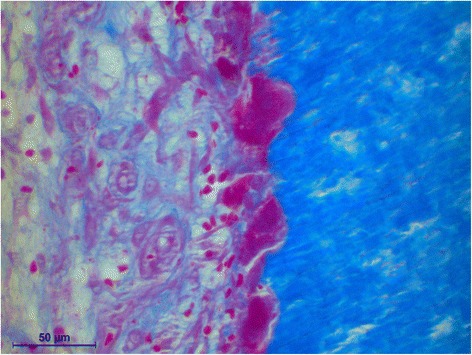
Higher magnification of the area with chronic inflammation, exhibiting fibrous sheets around the blood vessels

**Fig. 8:**
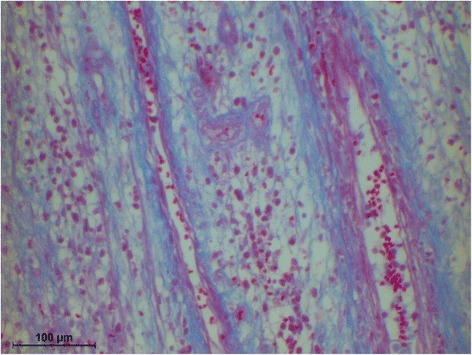
Higher magnification of the root pulp tissue, showing mild leucocyte infiltration

**Fig. 9:**
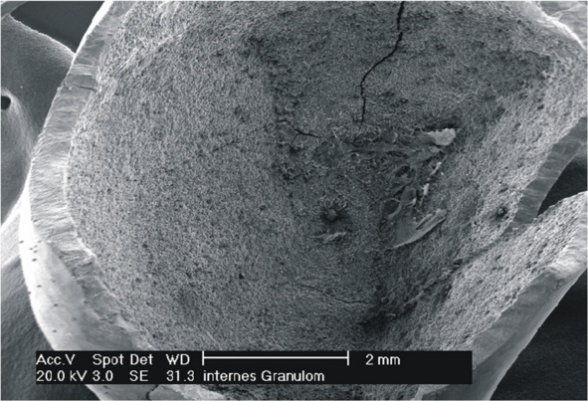
SEM picture of the inner side of the crown, showing extensive enamel and dentin resorption

**Fig. 10:**
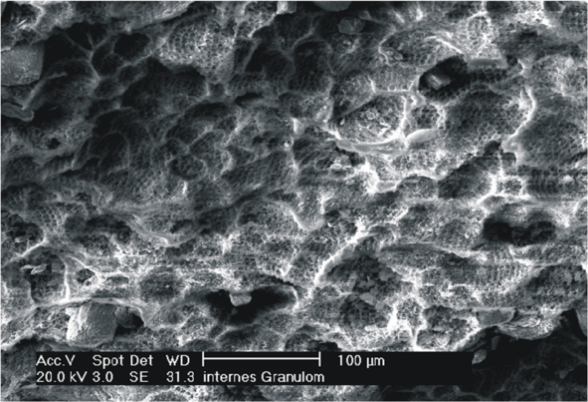
SEM picture of enamel resorption, with typical keyhole pattern and deep resorption lacunae

**Fig. 11:**
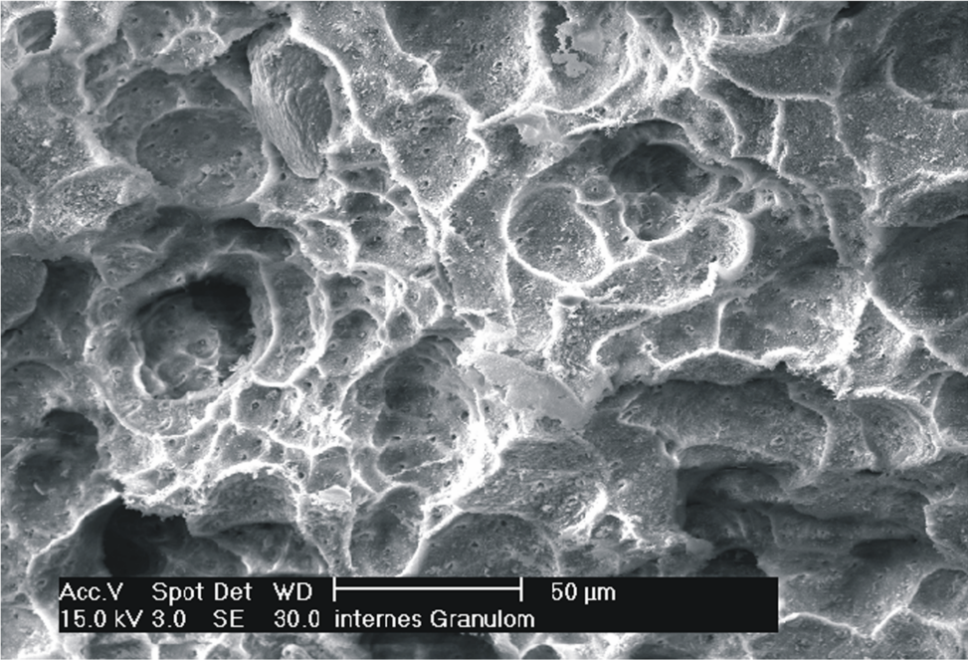
SEM picture of dentin resorption, with open dentin tubules and resorption lacunae

The histology of the granuloma showed four characteristic zones. The first zone showed tissue necrosis within the upper third of the crown pulp (Fig. 3), resulting in clinically dark discoloration. The second zone was identified as an area of exacerbated inflammation with micro-abscesses (Fig. 4). In the lower two thirds of the pulp tissue a third zone, highly vascularized tissue with polymorph nuclear infiltration of inflammatory cells was found (Fig. 5). The blood vessels were surrounded by fibrous tissue indicating chronic inflammation (Fig. 6). At the pulp dentin border, numerous polynuclear odontoclasts were found within the resorption lacunae (Fig. 7). In the fourth zone, the root pulp demonstrated an approximately normal histology with little leucocyte infiltration (Fig. 8), indicating that the tooth was still vital, though it was no longer reacting sensibly in this case. An SEM study of the coronal part of the tooth showed deep resorption lacunae (Fig. 9) within the enamel and dentin. A typical keyhole structure was observed within the enamel (Fig. 10), whereas open dentin tubules were identified within the areas of resorbed dentin (Fig. 11).

## Discussion

Internal root resorption is a rare remodeling process [3] that is mostly described within case reports in the literature [2–4, 7]. On the basis of a light microscopic study of the whole tooth root containing internal granuloma, it was our intention to look at resorption patterns resulting from different pathological, inflammatory and physiological processes using SEM. Several case reports of total or partial resorption of roots and crowns by exfoliating or retained neighboring teeth have been reported [8–10]. It has been concluded that hard tissue resorption of permanent teeth due to exfoliating neighboring teeth is caused by direct contact between these teeth and the cellular activities next to the contact points [11]. Resorption of retained or impacted third molars is ascribed to the loss of reduced ameloblastic epithelium, which is followed by odontoclasts. As a further consequence, ankylosis occurs as a response to resorption [11]. Odontoblasts and cementoblasts differ from osteoblasts in so far as they do not respond to hormones and cytokines that stimulate bone resorption. Hammarstrom and Lindskog [12] therefore concluded that the cementoblast or odontoblast layer has to undergo destruction due to necrosis or inflammation before dental hard tissue resorption is about to occur. Thus, it is concluded that there have to be further substances probably expressed by the reduced ameloblastic epithelium that make resorption of deciduous teeth possible. However, it becomes obvious why inflammation in case of periodontitis apicalis, and marginalis or trauma as an etiological factor of internal granuloma result in the resorption of dental hard tissues.

Concerning ultrastructural features of odontoclasts resorbing enamel, it could be demonstrated that these tartrate-resistant acid phosphatase-positive multinucleated cells were quite similar to the cells resorbing dentine or cementum [13]. Rich in mitochondria, lysosomes and free polysomes, these cells secrete acids, organic components and hydrolytic enzymes performing phagocytosis of enamel crystals then to be found in large cytoplasmic vacuoles to be dissolved intra-cellularly. From a cell biological standpoint, this should be the same ongoing process in enamel resorption performed by the resorptive tissue of internal granulomas.

Cytochemical studies revealed that H+ and K+ ATP-ase of odontoclast origin facilitate extracellular demineralization of inorganic crystals and hydrolytic enzymes, such as trimetaphosphatase and p-nitrophenyl phosphatase, and are responsible for intra- and extracellular digestion of organic components of the dentin and enamel, such as collagenous fibers [14]. Several studies have concentrated on resorption mechanisms due to molecular biological aspects, which were found with 6.9-fold enhanced DNA synthesis in mesenchymal cells, and 3.4-fold enhanced in epithelial cells gained from tissue resorbing deciduous teeth due to the application of epidermal growth factor (EGF) [15]. Using a dental hard tissue resorptive model, it has been shown that the lysosomal membrane antibody ED1 is a positive periodontal ligament cell (PDL) marker for mono- and multinucleated cells being involved in hard tissue resorption [16].

Lossdörfer et al. [17] assume that the receptors activator of nuclear factor kappa B /and ligand (RANK/RANKL), which are cytokine-like proteins of the TNF-family, play central roles in both physiological and pathological resorption. Odontoblasts, pulp fibroblasts, PDL-fibroblasts and odontoclasts were RANKL positive, and RANK positive immunostaining was also detectable in multinucleated odontoclasts, as in mononucleated precursors.

## Conclusions

Our results suggest that internal granuloma causes hard dental tissue resorption under pathological conditions, leading to different morphological patterns in dentin and enamel. Internal granuloma leads to irregular, distinct, shallow, but rigorous resorption patterns that could be demonstrated by SEM investigation. Light microscopy revealed 4 zones within the internal granuloma, namely, 1. necrosis, 2. areas of exacerbated inflammation with micro-abscesses, 3. chronic inflammation, and 4. an apically located zone of unaffected vital tissue of the root pulp.

## Consent

Written informed consent was obtained from all patients treated in the dental clinics of Witten/Herdecke University for publication of case reports and any accompanying images. A copy of the written consent is available for review at Witten/Herdecke University School of Dentistry.
